# Bcl-2 Family Proteins Are Involved in the Signal Crosstalk between Endoplasmic Reticulum Stress and Mitochondrial Dysfunction in Tumor Chemotherapy Resistance

**DOI:** 10.1155/2014/234370

**Published:** 2014-08-10

**Authors:** Jing Su, Lei Zhou, Mei-hui Xia, Ye Xu, Xi-yan Xiang, Lian-kun Sun

**Affiliations:** ^1^Key Laboratory of Pathobiology, Department of Pathophysiology, College of Basic Medical Sciences, Jilin University, Ministry of Education, Changchun 130021, China; ^2^Department of Pathology, Affiliated Hospital to Changchun University of Chinese Medicine, Changchun 130021, China; ^3^Department of Obstetrics, First Hospital, Jilin University, Changchun 130021, China; ^4^Medical Research Laboratory, Jilin Medical College, Jilin 132013, China

## Abstract

Tumor cells overexpress antiapoptotic proteins of the Bcl-2 (B-cell leukemia/lymphoma-2) family, which can lead to both escape from cell death and resistance to chemotherapeutic drugs. Recent studies suggest that the endoplasmic reticulum (ER) can produce proapoptotic signals, amplifying the apoptotic signaling cascade. The crosstalk between mitochondria and ER plays a decisive role in many cellular events but especially in cell death. Bcl-2 family proteins located in the ER and mitochondria can influence not only the function of the two organelles but also the interaction between them. Therefore, the Bcl-2 family of proteins may also be involved in the mechanism of tumor chemotherapy resistance by influencing crosstalk between the ER and mitochondria. In this review we will briefly discuss evidence to support this concept.

## 1. Introduction

The Bcl-2 (B-cell leukemia/lymphoma-2) family of proteins includes both antiapoptotic members (such as Bcl-2 and Bcl-xL) and proapoptotic members (multi-Bcl-2 homology- (BH-) domain proteins such as Bax and Bak and also BH3-only proteins). Bcl-2 was the first identified antiapoptotic gene and is widely expressed by hematopoietic cells, epithelial cells, lymphocytes, nerve cells, and a wide variety of tumor cells [1]. Overexpression of Bcl-2 in tumor cells can produce tolerance to a variety of anticancer drugs and mediate escape from cell death. Based on this theory, a series of Bcl-2 inhibitors, including HA14-1, GX15-070, BI-33, ABT 737, and S1, were synthesized by mimicking BH3-only proteins to enable a breakthrough in the study of antitumor therapy [2]. These BH3-only protein analogs (or BH3 mimetics) can competitively combine with antiapoptotic proteins, including Bcl-2, Bcl-xL, Bcl-w, and Mcl-1, followed by the release of the proapoptotic proteins Bax and Bak, which ultimately induces apoptosis [3].

An increasing number of reports in the literature have confirmed that multiple organelles and genes are involved in the mechanism of tumor chemotherapy resistance [4, 5]. Two-way communication between endoplasmic reticulum (ER) and mitochondria regulates both physiological and pathological processes in cells including mitochondrial energy metabolism, lipid metabolism, Ca^2+^ signaling pathways, and cell death [6]. Studies have found that Bcl-2 proteins are distributed not only in the mitochondrial outer membrane, but also in the cell membrane, ER, and nuclear membrane [7]. Bcl-2 family proteins located in the ER are known to be involved in the regulation of endoplasmic reticulum signaling pathways [2]. Therefore research on the role of Bcl-2 family proteins in the delivery of proapoptotic signals to mitochondria via the ER can help us understand how tumor chemotherapy resistance develops.

Notably, the Bcl-2 family proteins are not only involved in apoptosis, but also participate in the unfolded protein response (UPR) and calcium balance in the ER and are a focal point in the complex signaling network present in cells. In this paper, we will focus on the regulatory role of Bcl-2 family proteins in the crosstalk between endoplasmic reticulum stress and mitochondrial dysfunction in tumor chemotherapy resistance.

## 2. Bcl-2 Family Proteins Are Involved in the ER-Mediated Pathway of Apoptosis

The ER is one of the most important membranous organelles in cells and is involved in many important cellular functions including protein folding and transportation, lipid synthesis, and maintaining calcium homeostasis [8]. The ER is the sensor for intra- and extracellular environmental stimuli and monitoring and maintaining cell homeostasis. ER provides a platform where environmental signals can connect to biological structure and function, enabling the integration of multiple stress signaling pathways [9]. The disruption of cell homeostasis will lead to ER dysfunction and gradually reduce the cell's ability to react to physiological stress. When unfolded or misfolded proteins accumulated in the ER lumina, an adaptive response called the unfolded protein response (UPR) occurs. The UPR can alleviate ER stress by reducing protein synthesis, promoting protein degradation, and producing chaperones to assist with protein folding. Excessive or prolonged ER stress can lead to cell death mediated by apoptosis [10].

Chemotherapy resistance is often due to resistance of tumor cells to cell death induced by chemotherapeutic agents, which is the result of the loss of balance between cell survival and death. Existing research work shows that antitumor agents can induce ER stress, which then activates the UPR [11]. The UPR regulates gene transcription and translation through the IRE1/XBP1 (inositol-requiring enzyme 1/X-box binding protein-1), PERK/ATF4 (PKR-like ER kinase/activating transcription factor-4), and ATF6 (activating transcription factor-6) signal pathways. The ER is considered not only an early apoptosis checkpoint to reduce cell damage, but also an important participant in the amplification of the apoptosis signaling cascade. ER stress can lead to apoptosis through the mitochondrial apoptosis pathway. The UPR plays different roles in the mechanisms of the chemotherapeutic resistance or antitumor therapy according to the stimuli, cell context, and so forth [12].

The balance between different ER-localized Bcl-2 family proteins regulates the activation of ER signaling pathways and thus cell survival. ER-localized antiapoptotic proteins such as Bcl-2 and Bcl-xL suppress a variety of apoptosis inducing stimuli, including ER-localized proapoptotic proteins Bax/Bak and various BH3-only proteins. Both ER and mitochondrial apoptosis signaling pathways can lead to Bax and Bak activation and apoptosis, although there are differences between the two apoptosis signaling pathways [13]. Previous studies have found that under different cellular stresses, Bax and Bak can directly combine with IRE1*α* on the cytoplasmic side of the ER membrane. IRE1*α* then recruits ASK1, leading to activation of the proapoptotic signaling kinase JNK and downstream proapoptotic transcription factors c-Jun and p38 MAPK. This causes upregulation of CHOP/GADD153 and caspase-4 activation [14]. CHOP/GADD153 is an ER stress-specific transcription factor, which belongs to the C/EBP transcription factor family. CHOP/GADD153 plays an important role as a switch in apoptosis. ASK1 and JNK activation can also phosphorylate Bcl-2 and the BH3-only protein Bim, resulting in inactivation of the antiapoptotic protein, promoting proapoptotic protein activation and induction apoptosis. ATF4 is also activated by the UPR and can directly bind the Mcl-1 promoter, regulating the transcription of Mcl-1 [15].

Our previous work based on the BH3-only protein mimics S1 and ABT-737 suggests that the antitumor activities of these two small molecules are related not only to the mitochondrial apoptosis pathway, but also to the ER stress-mediated pathway of apoptosis [4, 16]. ER-localized Bcl-2 family proteins play an important role not only in regulating apoptosis induced by ER stress but also in maintaining Ca^2+^ homeostasis. Abnormal Ca^2+^ release from ER is a critical early event in apoptosis [17, 18]. ER calcium-ATPase (SERCA) and inositol 1,4,5-trisphosphate receptor (IP3R) can regulate the processing of Ca^2+^ from the cytoplasm into ER and also the physiological transient release of Ca^2+^ back into the cytoplasm. Ca^2+^ release from ER during ER stress can also promote the migration of Bax to the mitochondrial membrane, resulting in Bax oligomerization in the mitochondrial outer membrane and cytochrome c [19]. Bcl-2 and Bcl-xL can affect IP3R in a variety of ways, thus reducing the endoplasmic reticulum Ca^2+^ capacity, leading to decreases in cytoplasmic Ca^2+^ and the mitochondrial Ca^2+^ response, thus inhibiting Ca^2+^-dependent cell death [20]. Furthermore, knockout of the proapoptotic genes Bax and Bak can also lead to a reduction in the endoplasmic reticulum Ca^2+^ capacity [21]. Thus, Bcl-2 family proteins can influence apoptosis through the regulation of intracellular Ca^2+^ homeostasis.

## 3. The Bcl-2 Family of Proteins Regulates Contact between ER and Mitochondria

ER and mitochondria are closely connected, both structurally and functionally. Recent studies have further confirmed that the connection between ER and mitochondria (endoplasmic reticulum-mitochondria contacts) is the foundation of cell organelle communication [22]. This two-way communication is involved in the regulation of a variety of physiological and pathological processes, including mitochondrial energy metabolism, lipid metabolism, Ca^2+^ signaling ER pathways, and cell death [23]. Mitochondria are connected to the endoplasmic reticulum at specific sites called mitochondrion-associated membranes (MAMs). MAMs are adjacent to the ER and contain important Ca^2+^ transport-related proteins including IP3R, VDAC, GRP75, Sig-1R, and Bip [24]. The changing of morphology, intracellular ATP, or Ca^2+^ concentration can all affect ER-mitochondria contacts; therefore all of these factors may also be involved in regulating two-way communication between mitochondria and ER.

Bcl-2 family proteins are involved in the regulation of mitochondrial fission and fusion and can also control the morphology of mitochondria, thus affecting signaling between mitochondria and ER. Recent studies found that mitochondria form a reticular structure similar to ER in most kinds of cells. Mitochondrial reticular structure undergoes dynamic changes due to the processes of mitochondrial fission and fusion, which are precisely controlled by a variety of proteins. Drp1, Fis1, Caf4p, and Mdv1p are involved in the regulation of mitochondrial division. Mfn1/2 and OPA1 control the fusion of mitochondrial outer or inner membranes, respectively [25]. The Bcl-2 homolog CED-9 can interact with the mitochondria fusion protein Mfn2 and induce mitochondrial fusion in* C. elegans*. Bcl-xL can inhibit the GTPase activity of the mitochondrial fission protein Drp1. In contrast, the proapoptotic proteins Bak and Bax promote mitochondrial fission mediated by Drp1 [26]. Overexpression of the Bcl-2 family proapoptotic protein Bax can promote Drp1-dependent mitochondrial fragmentation [27]. Bax and Bak promote mitochondrial fragmentation by stabilizing the interaction of Drp1 and mitochondria during apoptosis [28]. Studies have shown that Noxa and Puma also promote Drp1-dependent mitochondrial fragmentation [13]. In summary, Bcl-2 family proteins can affect the balance of mitochondrial fission/fusion and thus regulate the morphology and function of mitochondria.

Bcl-2 family proteins can indirectly affect ER-mitochondria contacts by interacting with mitochondrial fission/fusion proteins as outlined above. Studies have shown that Mfn2 is distributed not only in mitochondria, but also in the ER surface linked to mitochondria. At the particular sites where mitochondria are connected with ER, the mitochondrial outer membrane fusion proteins Mfn1 and Mfn2 can form homo- or heterodimers in the ER membrane [14]. In Mfn2-defficient fibroblasts, the distance between ER and mitochondria increased, and the morphology of both organelles is changed. Mitochondrial fusion proteins are essential in both mitochondria and ER to connect the two organelles [15]. Additionally, the capacity of mitochondria for uptake of Ca^2+^ decreases after ER Ca^2+^ release induced by inositol triphosphate (IP3) in Mfn2-deficient cells. This indicates that Mfn2 plays an important role in maintaining Ca^2+^ signaling between mitochondria and ER and also in the Ca^2+^ capacity of mitochondria [15, 29]. According to a new study, the crosstalk between ER and mitochondria in apoptosis is mediated by the mitochondrial fission proteins Drp1 and Fis1. Fis1 can form a complex with the ER protein Bap31, which is mainly located where ER is connected with mitochondria. Fis1-Bap31 compounds can provide a platform for recruiting and activating caspase-8 early in apoptosis. Bap31 is then cleaved, leading to increased Ca^2+^ release from ER and induction of cytochrome c release from mitochondria [30]. In addition to Bap31 cleavage, Bik located in ER has also been shown to cause apoptosis mediated by Ca^2+^, leading to Drp1-dependent mitochondrial fission and the release of cytochrome c [22, 31].

## 4. The Bcl-2 Family Proteins Involved in Autophagy

Recent research confirmed that Bcl-2 family proteins including Bcl-2, Bax, Bak, and Bik are not only involved in the regulation of apoptosis but also regulate autophagy, which plays an important role in cell survival and death. Autophagy is a highly conserved strictly regulated process involving the degradation of long-life proteins and damaged organelles. Autophagy promotes cell survival under various stress conditions including ER stress or oxidative stress [32]. Studies have shown that autophagy is an important part of the normal function of the ER [33, 34]. ER stress-induced autophagy plays a central role in alleviating stress and maintaining homeostasis. There are two main cellular degradation systems present in eukaryotic cells: the ubiquitin-proteasome and autophagy-lysosome systems. The ER is associated with both degradation processes. Autophagy induced by ER stress is an alternative process of ER-associated protein degradation (ERAD) to degradation of misfolded proteins in the ER lumen. Autophagy can also degrade damaged ER itself to maintain ER function and homeostasis.

The loss of mitochondrial membrane potential can lead to both the release of proapoptotic factors from mitochondria and the production of reactive oxygen species. Dysfunctional mitochondria will be selectively cleared by autophagy. The healthy mitochondria are mainly through the PINK1-mediated phosphorylation of kinases reaction and subsequent E3 ligase Parkin-mediated ubiquitin reaction to clear them [10].

Studies have found that the BH3-only protein mimics ABT-737 and HA14-1 can competitively inhibit ER-localized Bcl-2 and Bcl-xL and prevent Bcl-2/Bcl-xL combining with Beclin1 and the activation of Beclin1-dependent autophagy [35–37]. Moreover, BH3-only protein mimics can also regulate cell autophagy by other pathways [38]. Activation of the UPR signaling pathway PERK-eIF2*α*-ATF4 can upregulate the expression of the autophagy gene Atg12 [39]. The interaction of IRE*α* and Bax/Bak can also promote the activation of the JNK pathway during ER stress. Notably, the early period of JNK activation promotes autophagy, while continuous activation of JNK leads to apoptosis [40, 41].

S1 is a small-molecule mimic of the BH3-only protein Bid [42]. Studies based on two-dimensional nuclear magnetic and fluorescence polarization experiments show that S1 has a high affinity for Bcl-2 and Mcl-1. S1 can therefore interfere with the interactions of Bcl-2/Bax or Mcl-1/Bak, thus inducing apoptosis in various tumor cells [43], including liver and breast cancer [44]. Our recent study shows that S1 can also induce apoptosis in glioma U251 cells, accompanied by upregulation of the expression of the autophagy marker protein LC3-II and ER stress marker protein PDI. This indicates that ER stress and autophagy are involved in the antitumor effects of S1, in addition to apoptosis. Our research work suggests that S1 can also induce ER stress-mediated apoptosis [4, 16].

## 5. Conclusion

The findings detailed above demonstrate that Bcl-2 family proteins are a common link between ER stress, mitochondrial dysfunction, and autophagy. Further studies are required to confirm the regulatory role of Bcl-2 family proteins in the crosstalk between endoplasmic reticulum stress and mitochondrial dysfunction in tumor chemotherapy resistance. However, these data provide hope for the development of novel antitumor therapeutic strategies based on Bcl-2 inhibitors to overcome tumor chemotherapy resistance.
